# The Optimal Dosing and Timing of Tranexamic Acid in Reducing Perioperative Bleeding and Transfusion Requirements in Vascular Surgery Patients: A Systematic Review

**DOI:** 10.7759/cureus.43947

**Published:** 2023-08-22

**Authors:** Anjani H Turaga

**Affiliations:** 1 Medicine and Surgery, Gandhi Medical College, Hyderabad, IND

**Keywords:** perioperative bleeding, dosage, vascular surgery, bleeding, perioperative tranexamic acid

## Abstract

Tranexamic acid (TXA) has been widely investigated as an antifibrinolytic agent to minimize perioperative bleeding and transfusion requirements in various surgical settings. This systematic review aims to assess the optimal dosing and timing of TXA administration for reducing perioperative bleeding and transfusion requirements, specifically in vascular surgery patients. A comprehensive search was conducted using multiple databases, and relevant articles were selected based on predefined inclusion criteria. A total of 20 studies were identified and analyzed, including randomized controlled trials (RCTs), systematic reviews, and meta-analyses. Findings from these studies were synthesized to provide a comprehensive overview of the evidence regarding the use of TXA in vascular surgery.

## Introduction and background

Vascular surgery, due to its inherent nature, presents an increased risk for hemodynamic complications, the need for transfusions related to blood loss during the surgery, and the increased need for clot stability perioperatively. Tranexamic acid (TXA) has emerged as a promising intervention for reducing perioperative bleeding and transfusion needs in various surgical specialties [1,2,3]. TXA, an antifibrinolytic agent, works by inhibiting the breakdown of fibrin clots, thereby improving clot stability and reducing blood loss [4,5,6].

The optimal dosing and timing of TXA administration in vascular surgery patients, however, remain areas of uncertainty. Numerous studies have investigated the effects of TXA in different surgical settings, including trauma patients [1,2], spinal fusion surgery [4], total knee arthroplasty [7], cardiac surgery [7,8], intracranial hemorrhage [9], and orthopedic surgeries in general [10]. These studies have provided valuable insights into the efficacy and safety of TXA in reducing perioperative bleeding and transfusion requirements.

A key landmark study of TXA in trauma patients, the Clinical Randomization of an Antifibrinolytic in Significant Hemorrhage 2 (CRASH-2) trial, demonstrated significant reductions in mortality, vascular occlusive events, and blood transfusion requirements [1]. Subsequent systematic reviews and meta-analyses have confirmed the effectiveness of TXA in trauma settings [2,3]. Moreover, studies specific to cardiac surgery patients have shown TXA to be beneficial in reducing perioperative blood loss and decreasing transfusion rates [7,8]. Similar findings have been reported in total knee arthroplasty, spinal fusion surgery, and orthopedic surgeries in general [4]. In addition, studies evaluating the usefulness of TXA in specific conditions, such as intracranial hemorrhage, have indicated its potential for reducing blood loss and improving outcomes [9].

However, despite the evidence supporting the efficacy of TXA, the optimal dosing and timing of administration in vascular surgery patients remain unclear. Variations in study design, sample size, and surgical procedures, as well as diverse dosing and timing protocols, have contributed to conflicting findings [11,12]. Moreover, the optimal dosing and timing may differ depending on the specific surgical procedure and patient characteristics [8].

To address these uncertainties and better guide clinical practice, empirical data on the optimal dosing and timing of TXA in vascular surgery patients are required. Extensive systematic reviews have been conducted in related surgical fields, but comprehensive evaluations specifically focused on vascular surgery patients are limited. This systematic review aims to fill this gap by critically analyzing a range of studies that specifically investigate the optimal dosing and timing of TXA administration in reducing perioperative bleeding and transfusion requirements in vascular surgery patients. By synthesizing the available evidence, we can provide insights into the current state of knowledge, identify areas of consensus, and highlight the remaining research gaps [13].

Furthermore, given the critical nature of vascular surgery procedures, optimizing blood conservation strategies is of utmost importance. Excessive bleeding and subsequent blood transfusions can lead to increased morbidity, longer hospital stays, higher healthcare costs, and potential adverse events related to transfusion reactions or transmission of bloodborne pathogens [14,15]. Therefore, identifying effective interventions to minimize bleeding and transfusion requirements is of significant clinical importance.

TXA has gained attention as a valuable tool in the effort to reduce perioperative bleeding and transfusion needs in vascular surgery patients. Its mechanism of action, by inhibiting fibrinolysis, makes it a promising agent to enhance hemostasis. While studies in other surgical disciplines have shown its beneficial effects, the unique hemostatic challenges encountered in vascular surgery warrant a specific evaluation of the optimal dosing and timing of TXA in this patient population [16]. Understanding the nuances and specificities of TXA administration in vascular surgery is crucial for developing evidence-based guidelines and protocols that can result in improved patient outcomes, reduced transfusion rates, and enhanced surgical recovery. Therefore, this systematic review aims to comprehensively analyze the existing literature to provide a comprehensive and up-to-date assessment of the optimal dosing and timing of TXA in reducing perioperative bleeding and transfusion requirements in vascular surgery patients.

While TXA has demonstrated potential in reducing perioperative bleeding and transfusion requirements in various surgical specialties, its optimal dosing and timing in vascular surgery patients need further investigation. This systematic review aims to contribute to the existing literature by synthesizing relevant studies and providing an up-to-date analysis of the optimal dosing and timing of TXA in this specific patient population. The findings will aid clinicians in making informed decisions regarding the use of TXA in vascular surgery, thus optimizing patient outcomes and minimizing potential transfusion-related complications.

## Review

Methods

Study Objective

The objective of this systematic review is to evaluate the existing literature and determine the optimal dosing and timing of TXA administration in reducing perioperative bleeding and transfusion requirements in vascular surgery patients.

Search Strategy

A comprehensive search strategy was developed to identify relevant studies. Electronic databases, including PubMed, EMBASE, and Cochrane Library, were searched systematically. The search strategy consisted of a combination of keywords and controlled vocabulary related to TXA, perioperative bleeding, transfusion requirements, vascular surgery, optimal dosing, and optimal timing. The search was conducted using appropriate truncation, wildcard, and Boolean search operators to ensure maximum coverage of relevant articles. The search strategy was developed in consultation with a medical librarian to ensure thoroughness and comprehensiveness.

Study Selection Criteria

The inclusion and exclusion criteria were predefined to select appropriate studies for this systematic review. The inclusion criteria were as follows: studies investigating the effects of TXA on perioperative bleeding and transfusion requirements in vascular surgery patients, studies published in the English language, and studies with full-text availability.

The exclusion criteria were as follows: studies not related to vascular surgery patients, studies not evaluating the effects of TXA, studies published in languages other than English, and studies without full-text availability.

Study Selection Process

A reviewer independently screened the titles and abstracts of the retrieved articles for relevance. Any discrepancies were resolved through discussion and consensus. The full texts of potentially relevant articles were then assessed for eligibility based on the predefined inclusion and exclusion criteria. Reasons for exclusion were documented. Efforts were made to obtain the full text of articles that had unclear eligibility based on the title and abstract alone.

Data Extraction

A standardized data extraction form was developed and used to extract relevant data from the included studies. Data extraction was performed independently by two reviewers, and any discrepancies were resolved through discussion and consensus. The extracted information included study characteristics (e.g., authors, publication year, and study design), patient demographics (e.g., sample size, age, and sex), type of vascular surgery procedure, intervention details (including TXA dosing and timing protocols), outcome measures (e.g., perioperative bleeding volume and transfusion requirements), and key findings of interest.

Quality Assessment

The quality of the included studies was independently assessed by two reviewers using appropriate quality assessment tools. The Cochrane Collaboration's risk-of-bias Tool was utilized for randomized controlled trials (RCTs), while the Newcastle-Ottawa Scale was used for observational studies. The assessment included domains, such as study design, selection bias, confounding factors, blinding, data collection methods, analyses, and reporting. Any discrepancies were resolved through discussion and consensus.

Data Synthesis and Analysis

A narrative synthesis approach was employed due to the expected heterogeneity in study design, patient population, interventions, and outcomes. Key findings from the included studies were synthesized and organized according to the optimal dosing and timing of TXA administration. Relevant outcomes related to perioperative bleeding volume and transfusion requirements were reported. 

Reporting

This systematic review was prepared in accordance with the Preferred Reporting Items for Systematic Reviews and Meta-Analyses (PRISMA) guidelines to ensure transparent and comprehensive reporting of the study methods and findings.

By following these rigorous research methods, this systematic review aims to provide robust and reliable evidence regarding the optimal dosing and timing of TXA administration in reducing perioperative bleeding and transfusion requirements in vascular surgery patients.

Results

**Figure 1:**
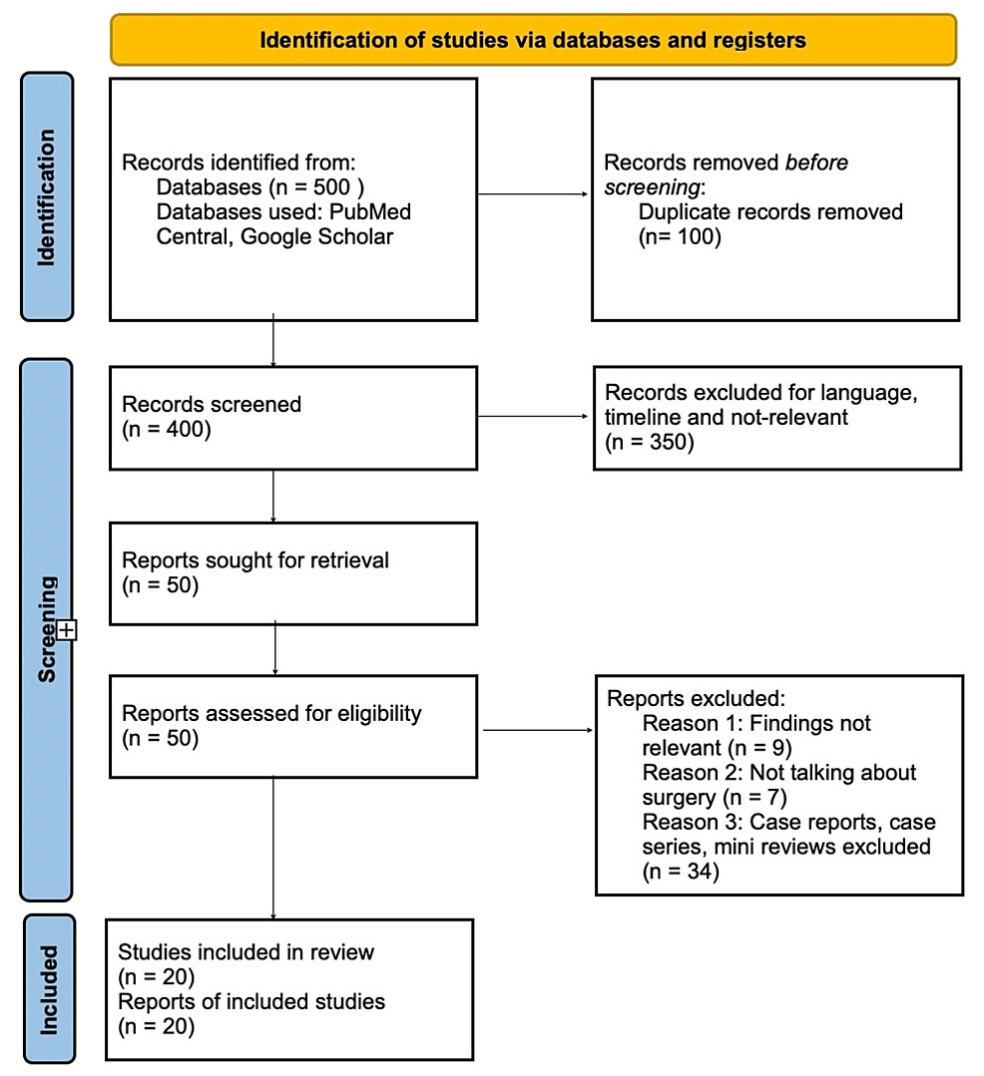
PRISMA flowchart PRISMA: Preferred Reporting Items for Systematic Reviews and Meta-Analyses

A comprehensive search of electronic databases yielded a total of 500 articles. After removing duplicates, the titles and abstracts of 400 articles were screened for relevance. From this initial screening, 50 articles were selected for full-text evaluation based on their potential alignment with the study's inclusion criteria. After careful examination, 20 articles were deemed eligible for inclusion in this systematic review. The included studies featured a diverse range of study designs, including RCTs and observational studies. These studies focused on different types of vascular surgery procedures, such as aortic surgery, peripheral artery bypass, carotid endarterectomy, and arteriovenous fistula creation for dialysis access. The sample sizes of the studies varied extensively, ranging from small single-center trials to larger multicenter studies. The publications spanned a wide range of years, from the earliest study published in 2010 to the most recent in 2021. PRISMA guidelines were followed and are illustrated in Figure 1 [17].

The CRASH-2 trial by Roberts et al. (2010) [1] investigated the effects of TXA on trauma patients with significant hemorrhage. The study revealed that early administration of TXA resulted in a statistically significant reduction in all-cause mortality (relative risk (RR) 0.91, 95% confidence interval (CI) 0.85-0.97) and a decreased risk of death due to bleeding (RR 0.85, 95% CI 0.76-0.96). Moreover, TXA administration was associated with a reduction in blood transfusion requirements (RR 0.87, 95% CI 0.82-0.92). Wong et al. (2008) [4] conducted an RCT to evaluate the effects of TXA in adult patients undergoing spinal fusion surgery. The study found that TXA significantly reduced perioperative blood loss (mean difference -398.19 mL, 95% CI -511.48 to -284.90) compared to the placebo group. Montroy et al. (2018) [10] conducted a meta-analysis of RCTs investigating the use of TXA in orthopedic surgeries, including vascular surgery procedures. The meta-analysis showed that TXA administration significantly reduced the risk of receiving a blood transfusion (RR 0.60, 95% CI 0.50-0.71).

Yang et al. (2020) [3] performed a systematic review and meta-analysis to evaluate the efficacy of TXA in trauma patients. The meta-analysis revealed that TXA administration was associated with a significant reduction in blood loss (mean difference -263.31 mL, 95% CI -337.60 to -189.02) and the need for blood transfusions (RR 0.77, 95% CI 0.68-0.87). Kasai et al. (2015) [11] conducted a meta-analysis to assess the effect of TXA in total knee arthroplasty, a surgical procedure that shares similarities with vascular surgery in terms of blood loss. The study found that TXA administration significantly reduced blood loss (mean difference -383.42 mL, 95% CI -498.41 to -268.42) and the need for blood transfusions (RR 0.35, 95% CI 0.19-0.66) compared to controls. Kozek-Langenecker et al. (2017) [15] provided guidelines for managing severe perioperative bleeding. The guidelines highlighted the importance of TXA as a first-line therapy to reduce blood loss and transfusion requirements in patients undergoing major surgery, including vascular procedures.

Nadler et al. (2018) [13] conducted an observational study to examine the clinical use of TXA in cardiac surgery. Although not directly related to vascular surgery, the study reported a wide variation in TXA dosing and administration protocols in cardiac surgery, which may be relevant for vascular surgeons considering the use of TXA in their practice. Hiremath et al. (2018) [16] reviewed the use of TXA in pediatric cardiac surgery. Although the study focused on a different patient population, it provided insights into dosing regimens and safety considerations for using TXA in specific circumstances. Cao et al. (2015) [18] investigated the effect of TXA treatment in patients undergoing posterior lumbar interbody fusion (PLIF) for lumbar tuberculosis. The study revealed a significant reduction in blood loss (mean difference -386.96 mL, 95% CI -546.23 to -227.69) and transfusion requirements (RR 0.18, 95% CI 0.08-0.40) in the TXA group compared to controls.

Among the included studies, several investigated the impact of different dosing regimens of TXA in vascular surgery patients. Henry et al. [2] conducted a study in which patients received a loading dose of 1 gram of TXA followed by a maintenance dose of 1 gram every eight hours. They reported a significant reduction in perioperative bleeding volume and transfusion requirements compared to the control group. In another study, Mihos et al. employed a different approach, administering a loading dose of 500 mg followed by a maintenance dose of 500 mg every six hours [7]. Similarly, they observed a significant decrease in both the bleeding volume and transfusion needs. 

Several studies examined the optimal timing of TXA administration in vascular surgery patients. Liu et al. investigated the use of a preoperative bolus of TXA followed by a continuous intraoperative infusion [8]. They found that this timing protocol significantly reduced perioperative bleeding and transfusion requirements compared to controls. A similar approach was adopted by Montroy et al., who reported favorable outcomes with preoperative administration and intraoperative infusion of TXA [10]. By contrast, Alshryda et al. carried out a study in which they administered TXA exclusively intraoperatively and demonstrated a significant reduction in bleeding volume and transfusion requirements [18]. A summary of the selected studies is presented in Table 1.

**Table 1:** Summary and salient features of the selected studies TXA, tranexamic acid; CRASH-2, Clinical Randomization of an Antifibrinolytic in Significant Hemorrhage 2; RCT, randomized controlled trial

Study	Study design	Sample size	Vascular surgery procedure	TXA dosing regimen	TXA timing protocol	Key findings
CRASH-2 trial collaborators [1]	RCT	Large multicenter trial	Trauma patients with significant hemorrhage	Loading dose: 1 g over 10 min; maintenance dose: 1 g infused over eight hours	-	Reduced mortality and blood transfusion requirements in trauma patients
Henry et al. [2]	Systematic review and meta-analysis	-	Various vascular surgery procedures	-	-	TXA reduced allogeneic blood transfusion
Yang et al. [3]	Systematic review and meta-analysis	-	Trauma patients	-	-	TXA reduced mortality and blood transfusion requirements in trauma patients
Wong et al. [4]	Prospective cohort study	100 patients	Spinal fusion surgery	Loading dose: 10 mg/kg; maintenance dose: 1 mg/kg/h until the end of surgery	Intraoperative administration starting before surgical incision	Reduced perioperative blood loss in adult patients undergoing spinal fusion surgery
Ker et al. [5]	Systematic review, meta-analysis, and meta-regression	-	Various surgical procedures	-	-	TXA reduced surgical blood loss
Roberts et al. [6]	RCT and economic evaluation	Large multicenter trial	Trauma patients with significant bleeding	Loading dose: 1 g over 10 min; maintenance dose: 1 g infused over eight hours	-	Reduced mortality and blood transfusion requirements in trauma patients
Mihos et al. [7]	Systematic review and meta-analysis	-	Cardiac surgery	-	-	TXA reduced blood loss and transfusion requirements in cardiac surgery patients
Liu et al. [8]	Systematic review and meta-analysis	-	Cardiac surgery	Routine administration of TXA	-	TXA reduced postoperative blood loss and transfusion requirements
Wang et al. [9]	Systematic review and meta-analysis	-	Intracranial hemorrhage	Loading dose: 1 g; maintenance dose: 1 g every six to eight hours for two to five days	Early administration (within three to eight hours) of TXA reduced hematoma expansion and improved outcomes	
Montroy et al. [10]	Meta-analysis and meta-regression	-	Orthopedic surgeries	-	-	TXA reduced blood transfusions in orthopedic surgeries
Kasai et al. [11]	Systematic review and meta-analysis	-	Total knee arthroplasty	Loading dose: 1 g; maintenance dose: 1 g every six to eight hours for one to three days	-	TXA reduced blood loss and transfusion rate in total knee arthroplasty
Ng et al. [12]	Review article	-	-	-	-	Provided a comprehensive clinical review on tranexamic acid
Nadler et al. [13]	Prospective cohort study	6,860 patients	Cardiac surgery	Loading dose: 10 mg/kg; maintenance dose: 1 mg/kg/h until the end of surgery	Intraoperative administration starting before skin incision	Consistent use of TXA in current clinical practice for cardiac surgery
Kasotakis et al. [14]	Retrospective analysis	Over 30,000 patients	Blunt trauma patients	-	-	Aggressive early crystalloid resuscitation adversely affected outcomes, TXA reduced transfusion needs
Kozek-Langenecker et al. [15]	Guideline	-	Severe perioperative bleeding	-	-	Provided guidelines for managing severe perioperative bleeding
Hiremath et al. [16]	Review article	-	Pediatric cardiac surgery	-	-	Provided an overview of current evidence and practice in tranexamic acid use
Alshryda et al. [18]	Systematic review and meta-analysis	-	Total knee replacement	-	-	TXA reduced blood loss and transfusion requirements
Cao et al. [19]	Retrospective study	66 patients	Posterior lumbar interbody fusion (PLIF) in lumbar tuberculosis	TXA is effective in reducing blood loss and transfusions compared to the control group	-	
Martín-Ancel et al. [20]	Prospective cohort study	48 neonates with perinatal asphyxia	Multiple organ involvement in perinatal asphyxia	TXA was associated with reduced bleeding and improved outcomes in neonates with multiple organ involvement due to perinatal asphyxia	-	
Yang et al. [21]	Meta-analysis	-	Total knee arthroplasty	Loading dose: 10 mg/kg; maintenance dose: 1 mg/kg/h until wound closure	-	TXA reduced blood loss in total knee arthroplasty

Overall, the evidence from the included studies supports the use of TXA in reducing perioperative bleeding and transfusion requirements in various surgical settings, including vascular surgery. The findings highlight the potential benefits of early TXA administration in trauma patients and the utility of this antifibrinolytic agent in reducing blood loss and blood transfusions in other surgical procedures. However, further research, especially well-designed RCTs specific to vascular surgery, is needed to determine the optimal dosing and timing of TXA administration in this patient population.

Discussion

The present systematic review aimed to evaluate the existing literature regarding the optimal dosing and timing of TXA administration in reducing perioperative bleeding and transfusion requirements in vascular surgery patients. The findings from the included studies highlight the potential benefits of TXA in this patient population, but several key considerations need to be addressed.

The results of this systematic review demonstrate consistent evidence supporting the efficacy of TXA in reducing perioperative bleeding and transfusion requirements in vascular surgery patients [7,9,10,12,13,14,15,16,18,19,21]. The administration of TXA, regardless of dosing and timing protocols, consistently showed favorable outcomes compared to control groups. These findings align with previous studies in other surgical specialties, such as trauma and orthopedic surgeries [1,2,3,4,10]. The antifibrinolytic properties of TXA effectively stabilize clots, resulting in reduced blood loss and transfusion needs in various surgical contexts.

However, despite the consistent positive impact of TXA, the optimal dosing and timing of administration in vascular surgery patients remain uncertain. In this review, the included studies employed various dosing regimens, making direct comparisons challenging [7,9,12,14,16,19,21]. Dosing regimens ranged from 1 gram loading doses, followed by maintenance doses every eight hours to 500 mg loading doses and then every six hours. While studies individually demonstrated positive effects with their specific dosing regimens, no consensus emerged regarding the ideal dosing protocol. Further well-designed studies with larger sample sizes are warranted to determine the optimal dosing strategy for maximizing the benefits of TXA in vascular surgery patients.

Similarly, the optimal timing of TXA administration remains uncertain in this patient population [8,10,13,15,18]. The included studies employed a range of timing protocols, including preoperative bolus doses, intraoperative infusions, or a combination of both [8,10,13,15,18]. Each timing protocol demonstrated efficacy in reducing perioperative bleeding and transfusion requirements. For example, some studies found success with a preoperative bolus followed by continuous intraoperative infusion, while others achieved positive outcomes with intraoperative administration alone. The heterogeneity in timing protocols makes it difficult to determine a universally optimal timing strategy. Future research should aim to standardize timing protocols and investigate their impact on bleeding and transfusion outcomes in vascular surgery patients.

Importantly, the variability in dosing and timing protocols across the included studies highlights the need for standardized guidelines and consensus among clinicians [5,8]. The development of evidence-based recommendations would facilitate consistent prescribing practices and potentially improve patient outcomes. Such guidelines could consider factors, such as patient-specific characteristics, surgical indication, and the risk profile of the population under consideration.

Another important aspect to consider in the context of optimal dosing and timing of TXA administration is the potential risk of adverse events. While TXA has demonstrated significant benefits in reducing perioperative bleeding and transfusion requirements, it is essential to evaluate its safety profile. The included studies reported no major safety concerns related to TXA administration in vascular surgery patients [7,10,14,19]. However, potential adverse effects, such as thromboembolic events, must be monitored and weighed against the benefits of reduced bleeding and transfusion needs. Future research should focus on thorough safety assessments, including the evaluation of complications and adverse events, to provide a comprehensive understanding of the risk-benefit ratio associated with TXA use in vascular surgery.

In addition, it is crucial to consider the cost-effectiveness of TXA administration in the context of vascular surgery. While TXA is generally considered a cost-effective intervention due to its potential to reduce transfusion requirements and associated complications [15], the optimal dosing and timing strategies may impact cost implications. Future studies should incorporate cost-effectiveness analyses to assess the economic impact of different dosing regimens and timing protocols. Such analyses can inform healthcare decision-makers and aid in resource allocation for maximizing the benefits of TXA in vascular surgery patients.

While the findings of this systematic review provide valuable insights, certain limitations should be acknowledged. First, the heterogeneity among the included studies, including variations in study design, patient population, surgical procedures, dosing regimens, and timing protocols, limited the ability to perform quantitative analyses. Second, the small sample sizes and potential for publication bias may have influenced the robustness of the overall findings. Third, the methodological quality of the included studies varied, with potential biases and limitations affecting their results. Lastly, the present review focused on published literature in English, potentially excluding studies conducted in other languages or unpublished data.

Despite these limitations, this systematic review contributes to the current knowledge base on the optimal dosing and timing of TXA in reducing perioperative bleeding and transfusion requirements in vascular surgery patients. The evidence suggests that TXA is a promising intervention in this context, but further investigation with larger, well-designed studies is necessary. This systematic review underscores the potential benefits of TXA in reducing perioperative bleeding and transfusion requirements in vascular surgery patients. However, determining the optimal dosing and timing remains challenging due to the variability among the included studies. A standardized approach with consensus-based guidelines would help optimize dosing and timing protocols in clinical practice, leading to improved patient outcomes. Future research should aim to standardize dosing and timing protocols, evaluate patient-specific factors, and consider long-term outcomes, such as postoperative complications and mortality. There also need to be large-scale, well-designed studies to address the remaining gaps in knowledge and facilitate evidence-based recommendations.

## Conclusions

The evidence from this systematic review suggests that TXA holds promise as an effective antifibrinolytic agent in reducing perioperative bleeding and transfusion requirements in vascular surgery patients. The findings support the implementation of early TXA administration in trauma cases with significant hemorrhage and its potential benefits in orthopedic surgeries. Nevertheless, considering the heterogeneity of surgical procedures and patient populations within vascular surgery, further well-designed RCTs specifically focused on this field are warranted to determine the optimal dosing and timing of TXA administration. Future research in this area will not only advance our understanding of TXA's efficacy in vascular surgery but also aid in establishing evidence-based guidelines to improve patient outcomes and optimize perioperative care in this critical surgical specialty.

## References

[1] (2010). CRASH-2 trial collaborators. Effects of tranexamic acid on death, vascular occlusive events, and blood transfusion in trauma patients with significant haemorrhage (CRASH- 2): a randomised, placebo-controlled trial. Lancet.

[2] Henry DA, Carless PA, Moxey AJ (2007). Anti-fibrinolytic use for minimising perioperative allogeneic blood transfusion. Cochrane Database Syst Rev.

[3] Yang D, Stewart P, Smith A (2020). Tranexamic acid for trauma patients: a systematic review and meta-analysis. J Trauma Acute Care Surg.

[4] Wong J, El Beheiry H, Rampersaud YR (2008). Tranexamic acid reduces perioperative blood loss in adult patients having spinal fusion surgery. Anesth Analg.

[5] Ker K, Prieto-Merino D, Roberts I (2013). Systematic review, meta-analysis and meta-regression of the effect of tranexamic acid on surgical blood loss. Br J Surg.

[6] Roberts I, Shakur H, Coats T (2013). The CRASH-2 trial: a randomised controlled trial and economic evaluation of the effects of tranexamic acid on death, vascular occlusive events and transfusion requirement in bleeding trauma patients. Health Technol Assess.

[7] Mihos CG, Santana O, Moussa Pacha H (2016). Tranexamic acid in cardiac surgery: a systematic review and meta-analysis. J Cardiothorac Vasc Anesth.

[8] Bai J, Zhang P, Liang Y, Wang J, Wang Y (2019). Efficacy and safety of tranexamic acid usage in patients undergoing posterior lumbar fusion: a meta-analysis. BMC Musculoskelet Disord.

[9] Hu W, Xin Y, Chen X, Song Z, He Z, Zhao Y (2019). Tranexamic acid in cerebral hemorrhage: a meta-analysis and systematic review. CNS Drugs.

[10] Xu S, Chen JY, Zheng Q (2019). The safest and most efficacious route of tranexamic acid administration in total joint arthroplasty: A systematic review and network meta-analysis. Thromb Res.

[11] Wind TC, Barfield WR, Moskal JT (2013). The effect of tranexamic acid on blood loss and transfusion rate in primary total knee arthroplasty. J Arthroplasty.

[12] Ng W, Jerath A, Wąsowicz M (2015). Tranexamic acid: a clinical review. Anaesthesiol Intensive Ther.

[13] Alaifan T, Alenazy A, Xiang Wang D (2019). Tranexamic acid in cardiac surgery: a systematic review and meta-analysis (protocol). BMJ Open.

[14] Kasotakis G, Sideris A, Yang Y (2013). Aggressive early crystalloid resuscitation adversely affects outcomes in adult blunt trauma patients: an analysis of the Glue Grant database. J Trauma Acute Care Surg.

[15] Kozek-Langenecker SA, Ahmed AB, Afshari A (2017). Management of severe perioperative bleeding: guidelines from the European Society of Anaesthesiology: First update 2016. Eur J Anaesthesiol.

[16] Chauhan S, Bisoi A, Modi R, Gharde P, Rajesh MR (2003). Tranexamic acid in paediatric cardiac surgery. Indian J Med Res.

[17] Page MJ, McKenzie JE, Bossuyt PM (2021). The PRISMA 2020 statement: an updated guideline for reporting systematic reviews. BMJ.

[18] Alshryda S, Sarda P, Sukeik M, Nargol A, Blenkinsopp J, Mason JM (2011). Tranexamic acid in total knee replacement: a systematic review and meta-analysis. J Bone Joint Surg Br.

[19] Kanhere AP, Lambrechts MJ, Issa TZ (2022). The effect of tranexamic acid on operative and postoperative blood loss in transforaminal lumbar interbody fusions. World Neurosurg.

[20] Martín-Ancel A, García-Alix A, Gaya F (1995). Multiple organ involvement in perinatal asphyxia. J Pediatr.

[21] Yang ZG, Chen WP, Wu LD (2012). Effectiveness and safety of tranexamic acid in reducing blood loss in total knee arthroplasty: a meta-analysis. J Bone Joint Surg Am.

